# Preventive Care for Non-Communicable Diseases in Greece: A Situational Analysis of Disease Burden, Service Delivery, Financing, Screening and Quality Governance

**DOI:** 10.3390/healthcare14183116

**Published:** 2026-09-21

**Authors:** Christos Triantafyllou, Anastasia Ntikoudi, Anastasia Papachristou, Vion Psiakis, Valter R. Fonseca, Joao Breda

**Affiliations:** WHO Athens Quality of Care and Patient Safety Office, 10675 Athens, Greece; ntikoudia@who.int (A.N.); papachristoua@who.int (A.P.); psiakisv@who.int (V.P.); fonsecav@who.int (V.R.F.); rodriguesdasilvabred@who.int (J.B.)

**Keywords:** non-communicable diseases, prevention, screening, primary health care, quality of care, health inequalities, Greece, public health policy

## Abstract

**Background:** Non-communicable diseases (NCDs) account for a substantial share of morbidity and mortality in Greece and create increasing demands on prevention, primary care, and long-term disease management. This study assessed the current national situation in NCD prevention, focusing on disease burden, service organization, financing, screening, surveillance, quality governance, and recent policy reforms. **Methods:** A structured narrative situational analysis was conducted using peer-reviewed literature, national statistics, administrative and policy documents, and reports from Greek and international organizations. Evidence available up to 10 August 2026 was synthesized across six domains: epidemiological burden, preventive-service delivery, financing, screening and early detection, surveillance and information infrastructure, and quality governance. No meta-analysis was undertaken. **Results:** In 2019, 41.7% of people aged ≥15 years reported a chronic illness or health problem. In 2023, circulatory diseases accounted for 31.9% of deaths and neoplasms for 23.5%. Cancer, cardiovascular diseases, diabetes, and chronic obstructive pulmonary disease account for an estimated 52% of premature deaths before age 75. Preventive expenditure represented 3.1% of health expenditure in 2023, while private payments accounted for 34.2% of total health expenditure in 2024. In 2019, reported screening participation was 66% for breast cancer, 73% for cervical cancer, and 28% for colorectal cancer. Organized prevention has subsequently expanded through the National Public Health Prevention Programme “PROLAMVANO,” with more than 5.2 million citizens participating in preventive examinations by February 2026. However, primary-care capacity, continuity, financial protection, follow-up after abnormal screening results, and longitudinal outcome measurement remain important challenges. **Conclusions:** Recent reforms indicate an expansion of organized and digitally enabled NCD prevention infrastructure in Greece, although their impact on primary-care integration, continuity of care, and population health outcomes remains uncertain. Future priorities include equitable completion of diagnostic and treatment pathways, sustainable financing, interoperable information systems, and routine measurement of population and patient-reported outcomes.

## 1. Introduction

Non-communicable diseases (NCDs) represent a major and growing health-system challenge in Greece. Cardiovascular diseases, cancer, chronic respiratory diseases, and diabetes mellitus constitute the core of the NCD burden and together account for an estimated 52% of premature deaths before the age of 75 [1]. Population aging, multimorbidity, and persistent exposure to modifiable risk factors are expected to increase future demand for prevention and chronic-disease management. OECD modeling suggests that, even if current risk-factor prevalence and survival remained unchanged, the number of NCD cases in Greece could increase by approximately 25% by 2050, while the number of people living with at least two concurrent NCDs could increase by 72% [1]. NCD occurrence and outcomes reflect the interaction of behavioral exposures, social and commercial determinants, environmental conditions, aging, and the capacity of health systems to prevent, detect, and manage disease at an early stage [1].

Greece presents a particularly important case for examining the transition from a curative and hospital-oriented model towards organized prevention. The national health system includes public-health authorities, primary-care structures, hospital outpatient departments, contracted professionals, municipalities, non-governmental organizations, and an extensive private ambulatory and diagnostic sector [2]. Although this pluralistic infrastructure creates multiple entry points for prevention, important access and financing challenges persist. In 2023, public financing accounted for approximately 61% of total health expenditure, while out-of-pocket payments represented 34%, more than twice the EU average of 16% [3]. Socioeconomic and geographical inequalities in access also remain important, reinforcing the need for prevention strategies that combine universal health coverage with targeted support for underserved populations [2,3].

The health policy environment has changed substantially in recent years. The National Prevention Public Health Program “Spiros Doxiadis” [4] established the framework for the expansion and modernization of population prevention, while the “Fofi Gennimata” breast-screening program demonstrated the feasibility of centrally coordinated, digitally enabled invitations [5]. This approach was subsequently expanded through the national “PROLAMVANO” program, incorporating organized preventive pathways for breast, cervical, and colorectal cancer, cardiovascular disease, renal dysfunction, and adult obesity [6,7]. In 2026, the Ministry of Health announced a new phase of PROLAMVANO extending from September 2026 to 2030 [8]. These national reforms have developed alongside major European initiatives, including Healthier Together, Europe’s Beating Cancer Plan, and the EU Safe Hearts Plan for cardiovascular health [9,10,11]. Collectively, these initiatives emphasize risk-factor reduction, organized early detection, digital prevention, quality assurance, stronger data systems, and reduction of health inequalities. Their success will depend not only on program reach, but also on participation, continuity of follow-up, sustainable financing, equity, outcome measurement, and institutional accountability [9,10,11].

Primary prevention, screening, early detection, and chronic-disease management represent related but distinct components of NCD control. Primary prevention aims to reduce disease occurrence through modification of behavioral, environmental, and metabolic risk factors, whereas screening and early detection seek to identify disease or elevated risk at an earlier stage [1,9,10,11,12]. Subsequent diagnostic confirmation, treatment initiation, and long-term risk-factor management form part of the broader continuum of NCD care. Importantly, increased screening activity or earlier detection should not in itself be interpreted as evidence of improved morbidity or mortality outcomes unless downstream diagnostic, treatment, and outcome indicators are also demonstrated [13].

Existing evaluations of the Greek health system have described important individual components of NCD prevention, including health-system organization, financing, disease burden, screening, primary-care capacity, and recent policy reforms [2,3,14,15,16]. However, these elements are generally reported across separate health-system assessments, statistical reports, disease-specific evaluations, and policy documents. The contribution of the present analysis is therefore primarily integrative: it brings together epidemiological burden, preventive-service delivery, financing, screening and early detection, surveillance and information infrastructure, and quality governance to examine how these components interact within the current Greek prevention landscape and to identify areas in which the expansion of preventive programs has not yet been matched by comprehensive outcome measurement [3,14,15].

The analytical structure was developed from the study objectives and informed by established health-system and NCD policy perspectives that emphasize the interaction between population health needs, service delivery, financing, information systems, governance, and prevention across the continuum of care [3,10,12]. Accordingly, six interrelated domains were defined: epidemiological burden and trends; organization and delivery of preventive services; financing and resource allocation; screening and early detection; surveillance and information infrastructure; and quality governance and reform. Together, these domains were intended to capture both the population-level burden of NCDs and the principal health-system functions required to support effective and equitable prevention.

This paper therefore synthesizes current evidence on NCD prevention and control in Greece with the aims of characterizing the epidemiological and economic burden of major NCDs, describing the organization and financing of preventive services, assessing screening and early-detection activity, identifying limitations in surveillance and quality governance, and outlining priorities for future evaluation and quality improvement within the Greek health system.

## 2. Methods

### 2.1. Study Design and Eligibility

This study employed a structured narrative situational-analysis design to examine the burden, organization, financing, delivery, surveillance, and quality governance of NCD prevention and control in Greece. This design was selected because the study required integration of heterogeneous evidence, including epidemiological statistics, population surveys, administrative data, policy and program documents, modeled estimates, and peer-reviewed research. The study was not designed as a systematic review or meta-analysis. Evidence identification, selection, and reporting were informed by methodological principles from contemporary scoping-review guidance and the PRISMA Extension for Scoping Reviews (PRISMA-ScR), while the overall design remained a structured narrative situational analysis. A predefined and structured process was used for evidence identification, eligibility assessment, data extraction, and narrative synthesis to improve transparency and consistency across the different evidence types.

The analysis was guided by the following question: What does the available evidence indicate regarding the burden, organization, financing, screening and early detection, surveillance, and quality governance of NCD prevention in Greece, and what limitations remain in the integration and evaluation of these activities?

The methodology of this situational analysis was retrospectively registered on the Open Science Framework (OSF; registration number: 10.17605/OSF.IO/FAS4E). The registration documents the study objectives, analytical domains, eligibility criteria, information sources, and approach to evidence synthesis.

Eligibility was structured according to the Population–Concept–Context framework. The population and geographic context comprised people residing in Greece and health-system, public-health, or policy evidence directly applicable to the Greek setting. Eligible concepts included NCD burden and modifiable risk factors, prevention and screening, early detection, primary-care organization, financing, surveillance and registries, equity, quality of care, and relevant national or European policies affecting NCD prevention and control in Greece.

Eligible evidence types included observational studies, population surveys, national statistics, administrative datasets, modeled epidemiological or economic estimates, government and institutional reports, program documents, policy reports, and publications from recognized national and international organizations. English- and Greek-language sources were eligible. No lower publication-date restriction was applied because historical policies and reforms were required to interpret changes over time; evidence available up to 10 August 2026 was considered.

Quantitative sources were required to provide sufficient information to identify the population or denominator, reference period, and provenance of the reported indicator. Institutional and policy sources were required to have an identifiable issuing organization, publication or reference date, and direct relevance to the Greek context. Sources were excluded if they focused exclusively on clinical treatment without relevance to prevention or health-system organization, consisted solely of unsupported opinion or commentary or duplicate or superseded reports, or provided insufficient methodological or contextual information to permit interpretation of the reported findings.

### 2.2. Information Sources and Search Strategy

Structured searches were conducted in PubMed/MEDLINE, Scopus, Web of Science Core Collection, and CINAHL. Because relevant health-system and policy evidence is frequently reported outside peer-reviewed journals, targeted searches were also undertaken across official sources, including the Greek Ministry of Health, ELSTAT, EODY, EOPYY, ODIPY, IDIKA/PROLAMVANO, WHO, OECD, Eurostat, the European Commission, the European Observatory on Health Systems and Policies, and European cancer-information resources.

Database searches were conducted separately in PubMed/MEDLINE, Scopus, Web of Science Core Collection, and CINAHL using combinations of the predefined geographical, NCD-related, and health-system/prevention concepts, adapted to the syntax and indexing structure of each database. English- and Greek-language terms were used where appropriate. The final database searches were completed by 10 August 2026. For institutional and grey-literature sources, targeted searches were conducted within the relevant official websites and document repositories using combinations of terms related to Greece, NCD prevention, screening, primary care, financing, surveillance, quality, and governance. Website searches were complemented by targeted browsing of relevant program, policy, and statistical sections and by reference-list searching.

Google Scholar and reference-list searching were used to identify additional relevant material. Searches included English- and Greek-language sources, with no lower date restriction because historical reforms and policies were required to interpret changes over time. Evidence available up to 10 August 2026 was considered. Search terms were organized into three principal concept blocks: geographical terms (“Greece” OR “Greek”); NCD-related terms (“non-communicable disease”, “NCD”, “chronic disease”, “cardiovascular disease”, “cancer”, “diabetes”, “chronic obstructive pulmonary disease”, “obesity”, and major modifiable risk factors); and health-system and prevention terms (“prevention”, “screening”, “early detection”, “primary care”, “financing”, “health expenditure”, “surveillance”, “registry”, “quality of care”, “governance”, “equity”, and “health inequality”). Terms were combined and adapted to the syntax and indexing conventions of each database and to the more focused search interfaces of institutional websites. The complete database-specific search strategies are provided in Supplementary Table S2.

Institutional and grey-literature sources were incorporated into the same overall evidence-selection framework as bibliographic records. Documents identified through official websites, Google Scholar, reference-list searching, and other targeted sources were screened for relevance and assessed against the same predefined eligibility criteria used for database-derived records. These sources were tracked separately by source category in the evidence-selection process to distinguish peer-reviewed bibliographic records from institutional, administrative, and policy documents.

### 2.3. Source Selection and Data Extraction

Bibliographic records were deduplicated and screened against the predefined eligibility criteria, followed by full-text assessment of potentially relevant sources. Official and institutional documents identified through targeted searches were assessed using the same eligibility principles.

Source screening and full-text eligibility assessment were performed independently by two reviewers. Any disagreements regarding eligibility were resolved through discussion and consensus, with consultation of a third reviewer where necessary. Data were extracted using a standardized source-level extraction framework that captured the source type, publication or reference year, population and denominator, data source or methodology, analytical domain, indicator definition, principal findings, measurement basis (e.g., self-reported, administrative, registry-based or modeled), and relevant methodological limitations.

**Figure 1:**
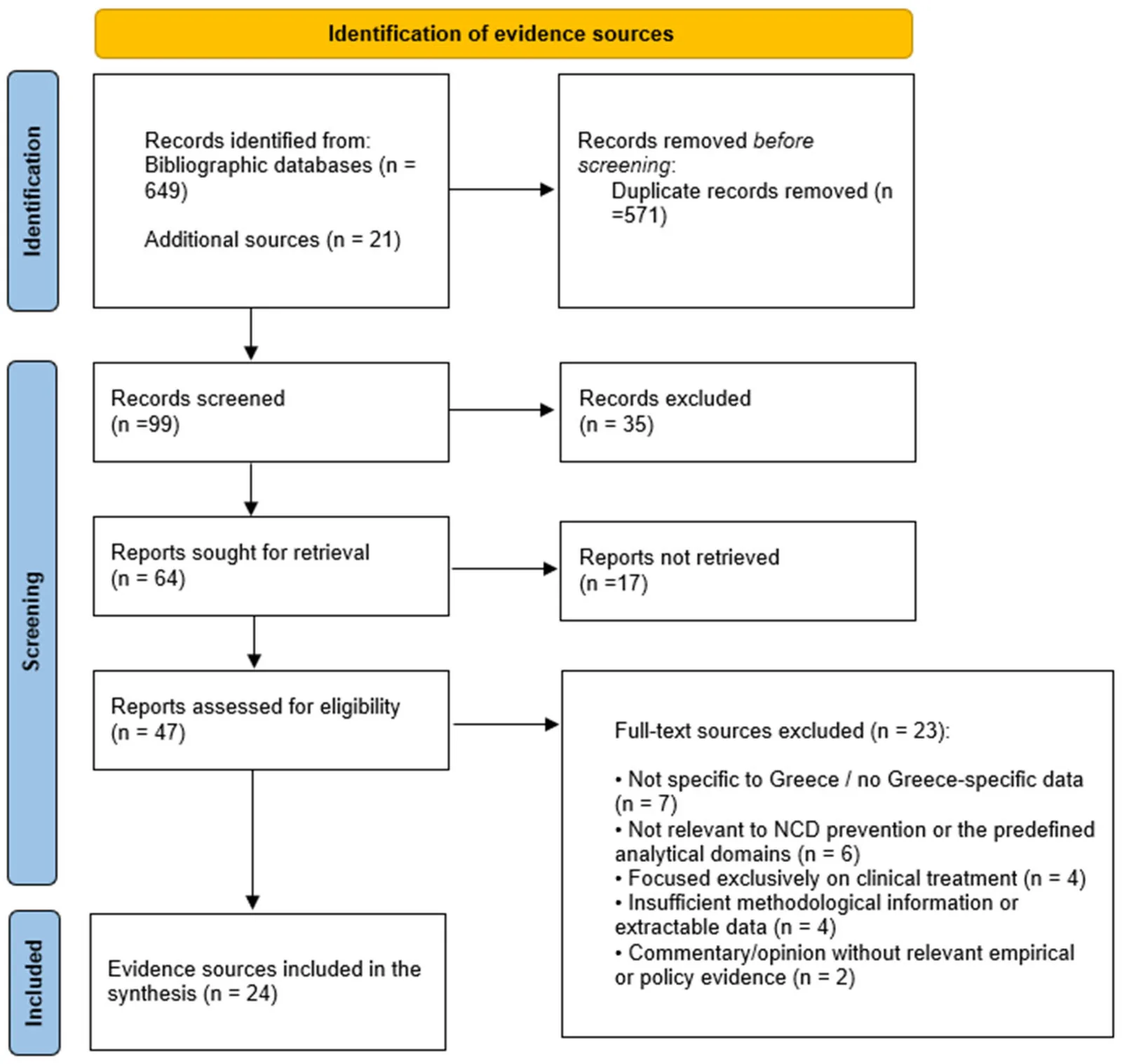
Flow diagram of evidence identification, screening, eligibility assessment, and inclusion, informed by PRISMA-ScR reporting principles.

Reasons for full-text exclusion were recorded, and the evidence-identification and selection process was summarized in a flow diagram (Figure 1).

### 2.4. Data Management and Synthesis

Where multiple publications reported the same underlying indicator, duplication was avoided, and preference was given to the most recent authoritative source with clear methodology and national coverage. National statistics were prioritized for nationally defined indicators and absolute counts, whereas OECD, Eurostat, and European Commission sources were used preferentially for standardized international comparisons.

Evidence was synthesized narratively across six domains: epidemiological burden and trends; organization and delivery of preventive services; financing and resource allocation; screening and early detection; surveillance and information infrastructure; and quality governance and reform. Survey-derived screening indicators were treated as self-reported participation measures and were not interpreted as equivalent to administrative program coverage.

No statistical weighting or pooling of heterogeneous evidence sources was undertaken. Modeled estimates were used when directly observed national data were unavailable or incomplete and were explicitly identified as modeled in the synthesis. They were interpreted according to their data provenance and methodological basis and were not treated as equivalent to directly observed national counts derived from vital statistics, population surveys, or administrative datasets. Direct temporal or international comparisons were made only when indicator definitions were sufficiently comparable. International comparisons were used descriptively and contextually and were not interpreted as evidence that specific organizational or financing models caused observed differences in health-system or population outcomes. Meta-analysis was not undertaken because of substantial heterogeneity in source types and measures.

### 2.5. Assessment of Evidence Quality

Formal risk-of-bias assessment was not undertaken because the evidence base comprised heterogeneous source types, including population surveys, administrative and statistical data, modeled estimates, policy documents, and program reports. Instead, sources were evaluated according to institutional authority, methodological transparency, national representativeness, data provenance, completeness, recency, and comparability. These characteristics were considered during interpretation of the findings, and conclusions based on more limited or indirect evidence were qualified accordingly. The absence of a formal design-specific critical-appraisal process is acknowledged as a limitation of the study.

## 3. Results

The searches identified 670 records from bibliographic databases and additional sources, including institutional and grey-literature searches. After deduplication and screening, 47 full-text sources were assessed for eligibility, of which 23 were excluded. A total of 24 evidence sources were included in the final synthesis, comprising peer-reviewed studies, national statistical sources, administrative and program documents, policy reports, and publications from national and international organizations (Figure 1).

The 23 excluded full-text sources were excluded because they lacked Greece-specific data, were not relevant to NCD prevention or the predefined analytical domains, had insufficient methodological information or extractable data, or consisted of commentary or opinion without relevant empirical or policy evidence (Figure 1).

The 24 included evidence sources comprised peer-reviewed studies, national statistical and administrative sources, program and policy documents, and reports from national and international organizations. Their characteristics and analytical contribution are summarized in Supplementary Materials Table S1.

### 3.1. Epidemiological Burden and Trends

National Health Interview Survey data indicate a substantial burden of chronic disease in Greece. In 2019, 41.7% of the population aged 15 years and over reported a chronic illness or health problem. Among specific conditions, 19.6% reported hypertension, 15.8% high blood lipids, 12.5% chronic low-back disorders, and 8.0% diabetes. Myocardial infarction was reported by 3.0% of the population, coronary heart disease or angina by 2.9%, and neoplastic diseases by 1.9% [17].

More recent estimates indicate 63,176 new cancer cases in 2022, corresponding to an age-standardized incidence rate of 529 per 100,000 population compared with 572 across the EU [14]. Prevalence rate (age-standardized) was higher, at 4814 per 100,000, compared with 4767 in the EU. Prostate and lung cancer predominated among men, while breast cancer accounted for one third of cases among women. Because Greece did not yet have a comprehensive population-based national cancer registry, these figures are modeled estimates rather than observed national registry counts [14].

Cancer mortality remained substantial, with an age-standardized rate of 239 per 100,000 in 2021, compared with 235 per 100,000 across the EU [14]. Between 2011 and 2021, cancer mortality declined by only 3.8% among men and 0.3% among women in Greece, compared with reductions of 16.1% and 8.5%, respectively, across the EU [14]. In 2023, diseases of the circulatory system accounted for 40,812 deaths (31.9% of all deaths), while neoplasms accounted for 30,095 deaths (23.5%) [18].

The broader NCD burden is also projected to increase. Cancer, cardiovascular diseases, diabetes, and COPD together account for an estimated 52% of premature deaths before age 75. OECD modeling projects an approximately 25% increase in NCD cases by 2050 and a 72% increase in the number of people living with at least two NCDs if current conditions persist [1]. Tobacco use, insufficient physical activity, obesity, and air pollution remain important modifiable contributors to this burden [1,19].

Recent national health-system assessment further indicates that NCDs account for approximately 85% of the total disease burden in Greece, while more than four in ten adults report a diagnosed chronic condition. Multimorbidity is particularly pronounced among older adults, with 52% of those aged over 65 reporting at least two chronic diseases [15].

### 3.2. Organization and Delivery of Preventive Services

Preventive care in Greece is delivered through a mixed public–private system involving health centers, primary-care structures, contracted physicians, hospital outpatient departments, and private diagnostic and ambulatory providers. The Ministry of Health retains responsibility for national health policy, while EOPYY acts as the principal purchaser of publicly financed healthcare. Although this pluralistic configuration provides multiple access points, fragmentation between providers and levels of care continues to affect continuity and coordination [3].

Recent reforms have strengthened the intended role of primary care in prevention and chronic disease management. The Personal Doctor scheme provides a defined point of first contact and is intended to improve navigation and continuity. However, workforce capacity remains limited: general practitioners account for approximately 6% of physicians compared with 21% across the EU, while nurse density is 3.8 per 1000 compared with 8.4 per 1000 across the EU [19]. These constraints limit the development of multidisciplinary and longitudinal NCD management [19].

PROLAMVANO has introduced a more proactive organizational model based on centrally identified eligible populations, electronic referrals, and access through participating public and private providers without direct payment. Initially focused on breast, cervical, and colorectal cancer screening, the program subsequently expanded to cardiovascular-risk assessment, renal dysfunction, and adult obesity [6,7].

By February 2026, more than 5.2 million citizens, corresponding to over 86% of approximately 6 million eligible beneficiaries, had reportedly participated in preventive examinations under PROLAMVANO, while almost 178,000 individuals had been identified with findings requiring further assessment across cancer and cardiovascular pathways [7]. These figures demonstrate the increasing scale of organized prevention, although screening and referral volumes alone do not establish program effectiveness or completion of subsequent care [7].

### 3.3. Financing and Resource Allocation

Total health expenditure reached EUR 19.87 billion in 2024, equivalent to 9.92% of GDP. Public funding represented 60.6% of total health expenditure, while private funding accounted for 39.1%, of which private payments represented 34.2% [20].

Preventive care nevertheless continues to receive a relatively small proportion of health-system resources. In 2023, preventive expenditure represented 3.1% of health expenditure in Greece, after temporarily increasing to 4.5% during the COVID-19 pandemic. Inpatient care continued to account for more than 40% of health expenditure compared with an EU average of 28%, illustrating the continuing hospital-oriented allocation of resources [15]. Although this was higher than pre-pandemic levels, part of the increase reflected immunization and other pandemic-related expenditure rather than structural investment specifically in NCD prevention [3].

The expenditure profile also remains treatment-oriented. Pharmaceuticals account for approximately 29% of health expenditure in Greece, compared with 19% across the EU, while inpatient care accounts for 42% compared with 28% across the EU [15]. Earlier disease-specific expenditure estimates showed that circulatory, digestive, neoplastic, endocrine, nutritional, and metabolic diseases together accounted for 48.5% of attributable current health expenditure in 2013 [21,22].

Country-specific OECD modeling further quantified the potential economic benefits of NCD prevention in Greece. Under a scenario in which risk-factor prevalence reached the levels observed in the top-performing quartile of countries, improvement in obesity alone was estimated to avert approximately 2177 premature deaths annually, reduce annual health expenditure by USD PPP 992.5 million (4.5%), and increase GDP by 0.9%, averaged over 2026–2050. Smoking and air pollution represented the next largest modifiable priorities in terms of projected health and economic gains [1]. These estimates are scenario-based model projections rather than observed program savings.

### 3.4. Screening and Early Detection

Historical screening estimates were survey-based and should not be interpreted as administrative program coverage. In 2019, 66% of women aged 50–69 reported mammography within the previous two years, 73% of women aged 20–69 reported cervical screening within the previous two years, and 28% of adults aged 50–74 reported colorectal-cancer screening within the previous two years [14]. These indicators therefore represent self-reported screening participation among the specified age-defined survey populations and pre-date the subsequent expansion of organized national screening programs.

Important inequalities were evident within these aggregate figures. Mammography was reported by 53% of women with lower educational attainment compared with 80% among those with higher education. Income, education, and country of birth were associated with differences in screening uptake, indicating that favorable national averages do not necessarily imply equitable access [14].

Organized national screening has expanded considerably through PROLAMVANO. The corresponding programs currently target approximately 1.8 million women aged 45–74 for breast-cancer screening, 2.5 million women aged 21–65 for cervical-cancer screening, and 2.8 million adults aged 50–69 for colorectal-cancer screening. These activities generated substantial numbers of referrals for additional diagnostic assessment [6].

Organized prevention subsequently expanded to cardiovascular risk, with free assessment introduced for adults aged 30–70 years. By February 2026, participation across PROLAMVANO had exceeded 5.2 million citizens [7]. However, program activity indicators should be distinguished from measures of effectiveness. Robust evaluation requires information on population coverage, positive-test rates, diagnostic confirmation and completion, time from abnormal screening result to diagnosis, treatment initiation, stage at diagnosis, interval cancers where applicable, subsequent morbidity and mortality, and inequalities in participation, diagnostic completion, treatment, and outcomes [6,7,14]. A recent national assessment similarly identified the absence of clearly defined and systematically monitored follow-up pathways after positive screening results, particularly for people in rural and underserved areas [15].

### 3.5. Surveillance, Registries, and Measurement of Quality

NCD-related information in Greece is generated through multiple population surveys, vital-statistics systems, administrative datasets, screening platforms, and disease-specific registries. The principal limitation is therefore not the existence of multiple information systems per se, but the limited routine linkage of these data sources across the full care pathway. Available evidence indicates that screening information is not yet systematically connected with primary-care, hospital, pathology, pharmaceutical, and mortality data in a way that permits routine longitudinal assessment from prevention and screening through diagnosis, treatment, and longer-term outcomes [3,15].

Cancer surveillance illustrates this limitation. Cancer surveillance has recently been strengthened, with the National Cancer Registry becoming operational in February 2025. Nevertheless, access to registry data remains limited, and comprehensive national information on stage at diagnosis, survival, recurrence, geographical variation, and socioeconomic inequalities is still developing [15].

These challenges should also be considered within the broader European context of fragmented oncology information systems. The Cancer Care Beacon project mapped cancer registries, administrative databases, and other oncology-related information resources across all 27 EU Member States and identified continuing challenges in data accessibility, organization, and interoperability. This European experience reinforces the importance of improving not only the availability of cancer data in Greece, but also their accessibility, standardization, and linkage across information systems [23].

The digital infrastructure created through PROLAMVANO provides an opportunity to improve population monitoring, because eligibility, examinations, findings, and referrals can now be recorded systematically. However, routine linkage with primary-care, hospital, pathology, laboratory, pharmaceutical, and mortality information remains necessary to support longitudinal evaluation of diagnostic completion, treatment, complications, and longer-term outcomes [6,7].

Quality measurement also remains limited for continuity, coordination, and patient-centered outcomes. Important quality indicators, including stage at diagnosis, diagnostic waiting times, 30-day mortality following acute myocardial infarction or stroke, and readmission rates for chronic conditions, are not yet systematically reported [15]. PROMs, PREMs, and quality-of-life measures are not yet routinely embedded across national NCD pathways. A more comprehensive monitoring architecture should therefore combine clinical and administrative indicators with patient-reported outcomes and stratify results by socioeconomic and geographical characteristics [3].

### 3.6. National Reform and Alignment with International Frameworks

The National Prevention Public Health Program “Spiros Doxiadis” has provided the principal policy framework for the recent expansion of prevention in Greece [4]. Under this framework, organized programs have extended from cancer screening to cardiovascular-risk assessment, renal-dysfunction detection, and adult-obesity management [6,7].

PROLAMVANO represents the principal operational expression of this shift towards centrally coordinated population prevention. In 2026, the Ministry of Health announced continuation of the program through a new phase extending from September 2026 to 2030, indicating an intention to establish a longer-term national preventive infrastructure [8]. Recent assessment has emphasized that several major preventive and digital reforms remain dependent on Recovery and Resilience Facility financing, making transition to stable long-term funding an important consideration for sustainability [15].

These reforms broadly align with the WHO NCD framework and the EU Healthier Together initiative through their emphasis on risk-factor reduction, early detection, surveillance, and equitable access [10,12]. Cancer screening also corresponds to the priorities of Europe’s Beating Cancer Plan, while the cardiovascular component increasingly aligns with the EU Safe Hearts Plan [9,11].

Overall, Greece has moved closer to international models of organized NCD prevention. The remaining issue is whether this expanding infrastructure can achieve sustained population coverage, complete diagnostic and treatment pathways, improved risk-factor control, and measurable reductions in preventable morbidity and mortality.

## 4. Discussion

### 4.1. Greece in Comparative Perspective

Greece shares with Italy, Spain, and Portugal the pressures associated with population aging, multimorbidity, and increasing demand for chronic-disease care, but its performance on avoidable mortality is less favorable. Preventable mortality is estimated at 141 deaths per 100,000 in Greece, compared with 117 in Portugal, 93 in Italy, and 92 in Spain, while treatable mortality is 72 per 100,000 compared with 63, 52, and 50, respectively [19]. These differences should be interpreted descriptively, as cross-country variation may reflect differences in population characteristics, service organization, financing, and measurement. Nevertheless, they provide important context for Greece’s continuing challenges in prevention, timely access to care, financial protection, and continuity. Recent reforms indicate convergence with broader European priorities towards organized prevention, initiatives intended to strengthen primary care, and digitalization, although important implementation and integration gaps remain [3,16].

### 4.2. Organized Prevention and Screening

PROLAMVANO represents an important convergence with European practice, where organized population-based breast, cervical, and colorectal cancer screening is increasingly standard. Recent reforms have expanded Greece’s screening approach beyond predominantly opportunistic provision through centrally defined eligibility, digital invitations, and publicly financed pathways. However, European experience demonstrates that the existence of an organized program does not necessarily ensure high or equitable participation. Portugal continues to report limitations in colorectal-screening uptake, while substantial regional variation persists within Italy; by contrast, countries such as Finland and the Netherlands demonstrate considerably stronger participation in organized colorectal screening [14,24].

Equity remains particularly important in Greece. Mammography was reported by 53% of women with lower educational attainment compared with 80% among those with higher education, indicating that universal eligibility does not eliminate socioeconomic barriers [14]. Similar inequalities are observed elsewhere in Europe, while approaches such as mobile services, home-based testing, community outreach, and primary-care involvement may support participation among underserved populations [24]. Evaluation of the next phase of Greek screening should therefore extend beyond invitations, tests, and referrals to population coverage, diagnostic completion, time to treatment, stage at diagnosis, and mortality.

### 4.3. Primary Care, Continuity, and Financing

Primary-care capacity remains an important constraint on effective NCD prevention in Greece. General practitioners account for approximately 6% of the physician workforce, compared with 21% across the EU, while nursing density is also comparatively low, although cross-country comparisons should be interpreted cautiously because Greek data include only hospital-based nurses [15,19]. Patient-reported evidence is consistent with these structural limitations: 47% of people with chronic conditions in Greece reported good care coordination, compared with 59% across participating PaRIS systems, while 43% of people with multiple chronic conditions reported being with the same primary-care professional for more than five years, compared with 58% on average [25]. Italy reported higher care coordination (72%), although the difference in non-physician involvement in chronic-disease management was smaller (69% in Italy versus 60% in Greece) [25]. These findings suggest that registration with a personal doctor alone is unlikely to ensure effective prevention without longitudinal, coordinated, and team-based care. This is consistent with a recent Greek NCD policy assessment identifying fragmented service organization, underdeveloped primary care, poor coordination, and workforce imbalances as persistent barriers to earlier intervention [15].

This is particularly relevant to cardiovascular prevention. Population screening can identify hypertension, dyslipidemia, obesity, and glycemic abnormalities, but detection alone is insufficient without sustained treatment and risk-factor management [13]. PROLAMVANO should therefore be linked closely with primary care and evaluated through subsequent risk-factor control and cardiovascular outcomes.

Financial protection represents a further challenge. Unmet healthcare need is approximately 12.1% in Greece, compared with 2.4% in Portugal, 1.8% in Italy, and 1.7% in Spain. In addition, only 61% of health expenditure in Greece is covered through mandatory prepayment, compared with 62% in Portugal and 73% in both Italy and Spain [19]. Free access to an initial screening test therefore does not necessarily guarantee equitable completion of diagnostic and treatment pathways when patients face financial or organizational barriers to follow-up.

### 4.4. From Program Activity to Measurable Outcomes

A central remaining challenge is the capacity to determine whether expanding preventive activity translates into better health outcomes and reduced inequalities. Countries with more mature NCD information infrastructures routinely use population-based registries and linked datasets to evaluate incidence, stage, survival, and complete care pathways. Greece continues to rely partly on modeled cancer-incidence estimates rather than complete population-based national registry counts, while routine linkage between screening, diagnosis, treatment, and outcomes remains limited [14].

Electronic prescribing and PROLAMVANO provide an important digital foundation for more comprehensive monitoring. Future evaluation should therefore move beyond invitations, examinations, and referrals towards population coverage, diagnostic confirmation, treatment initiation, time to diagnosis and treatment, stage at diagnosis, interval cancers where applicable, risk-factor control, preventable admissions, survival, preventable and treatable mortality, PROMs, PREMs, and inequalities in outcomes [15,25]. The key issue is therefore no longer only whether organized prevention is expanding, but whether the emerging infrastructure can demonstrate equitable and measurable improvements across the full prevention-to-outcome pathway.

## 5. Strengths and Limitations

The principal strength of this study is its integration of multiple complementary evidence types to provide a current system-level assessment of NCD prevention in Greece. The analysis incorporates national statistics, population surveys, administrative and program data, peer-reviewed research, public policy documents, and international comparative reports. The use of national statistical sources strengthens the description of mortality and health expenditure, while policy and program documents allow recent reforms to be considered alongside epidemiological and health-system indicators. The synthesis also distinguishes directly observed national statistics and self-reported survey indicators from administrative data and modeled estimates, thereby preserving important differences in data provenance and interpretation.

Several limitations should be acknowledged. First, although a structured search and predefined eligibility framework were used, targeted searching of institutional and grey-literature sources may have introduced selection bias. Second, no formal design-specific risk-of-bias assessment was undertaken; instead, sources were interpreted according to institutional authority, methodological transparency, national representativeness, completeness, recency, and comparability. Third, the evidence included was heterogeneous in design, reference year, population, and measurement method, and the evidence base was not uniformly distributed across the six analytical domains. Some areas, particularly surveillance, longitudinal outcome measurement, and quality governance, were supported by fewer national sources, which may limit the breadth of the conclusions that can be drawn in these domains. Some indicators were self-reported, others were administrative, and some cancer estimates were modeled, because comprehensive registry data remain incomplete. Recent program data mainly describe activity and referrals rather than independently evaluated downstream outcomes. Cross-country comparisons should also be interpreted cautiously because health systems differ in organization, financing, and data definitions.

## 6. Conclusions

Recent reforms indicate an expansion of organized and digitally enabled NCD prevention infrastructure in Greece, particularly through PROLAMVANO and policies intended to strengthen the role of primary care. These developments suggest a shift away from reliance solely on opportunistic prevention; however, the available evidence does not yet establish the extent to which this expanded infrastructure has strengthened primary care, improved continuity across diagnostic and treatment pathways, or translated into measurable improvements in population health outcomes. Important gaps remain in financial protection, primary-care capacity, continuity of care, equity, and longitudinal outcome monitoring.

The next phase should therefore prioritize program performance and consolidation rather than further program proliferation. Screening and risk assessment need to be integrated with primary care and closed-loop diagnostic, treatment, and follow-up pathways, supported by sustainable financing and interoperable national registries. Success should be assessed through equitable participation, risk-factor control, timely diagnosis and treatment, patient-reported outcomes, and reductions in preventable morbidity and mortality. A national NCD quality framework with transparent and routinely reported indicators could provide the accountability structure required to determine whether Greece’s expanding preventive infrastructure produces sustained and equitable population-health gains.

## Data Availability

No primary individual-level data were generated or analyzed in this study. The evidence synthesized in this analysis was extracted from publicly available sources cited in the manuscript. The source-level characteristics and analytical contribution of the included evidence are reported in Table S1.

## Supplementary Materials

The following supporting information can be downloaded at: https://www.mdpi.com/article/10.3390/healthcare14183116/s1, Table S1. Characteristics and analytical contribution of the evidence sources included. Table S2. Database search strategy.

## Abbreviations

The following abbreviations are used in this manuscript:

COPD	Chronic obstructive pulmonary disease
EOPYY	National Organization for Health Care Services Provision
EU	European Union
GDP	Gross domestic product
NCDs	Non-communicable diseases
OECD	Organisation for Economic Co-operation and Development
PaRIS	Patient-Reported Indicator Surveys
PREMs	Patient-reported experience measures
PROMs	Patient-reported outcome measures
WHO	World Health Organization
