# Supplementary material for: Preventive Care for Non-Communicable Diseases in Greece: A Situational Analysis of Disease Burden, Service Delivery, Financing, Screening and Quality Governance
Source: Healthcare (Basel). 2026 Sep 21;14(18):3116. doi: 10.3390/healthcare14183116 (PMC13606522; doi:10.3390/healthcare14183116)
Supplement: Supplementary file 1 [file healthcare-14-03116-s001.zip › healthcare-4547917-supplementary.pdf]

Table S1. Characteristics and analytical contribution of the evidence sources included

First author / issuing organization	Evidence type	Year / data basis	Analytical domain(s)	Main contribution to the synthesis
**OECD [1]**	International modelling report	2026; Greece-specific modelling and projections to 2050	Epidemiological burden; risk factors; economic burden	Quantifies premature mortality attributable to major NCDs and models future NCD burden and potential health and economic gains from reducing major risk factors.
**Economou et al. [2]**	Health-system review	2017; Greek health system	Service organization; primary care; financing; equity	Provides a comprehensive description of the organization, financing and performance of the Greek health system.
**OECD / European Observatory on Health Systems and Policies [3]**	Country health-system profile	2025; Greek health-system indicators	Financing; service delivery; prevention; access; system performance	Provides a current assessment of health status, risk factors, health-system organization and financing, accessibility, effectiveness and resilience in Greece.
**Ministry of Health, Greece [4]**	National policy document	National Action Plan for Public Health 2021–2025	Prevention policy; governance; reform	Provides the national policy framework underlying the recent expansion and modernization of public-health prevention in Greece.
**Ministry of Health, Greece [5]**	National programme source	“Fofi Gennimata” breast-cancer screening programme	Screening; early detection; service delivery	Documents the implementation of organized breast-cancer screening within the national

First author / issuing organization	Evidence type	Year / data basis	Analytical domain(s)	Main contribution to the synthesis
				prevention programme.
**Ministry of Health, Greece [6]**	National programme announcement	2025	Screening; cardiovascular prevention; service delivery	Documents the introduction of free cardiovascular-risk assessment within PROLAMVANO.
**Ministry of Health, Greece [7]**	National programme report	February 2026	Programme reach; screening; cardiovascular prevention; obesity; renal dysfunction	Provides programme participation data and documents expansion of PROLAMVANO to additional preventive pathways.
**Ministry of Health, Greece [8]**	National policy/programme announcement	2026; planned continuation September 2026–2030	Prevention reform; governance; sustainability	Documents announced continuation and further development of PROLAMVANO through 2030.
**European Commission [9]**	European policy framework	Europe's Beating Cancer Plan, launched 2021	Cancer prevention; early detection; quality; equity	Provides the European policy framework for cancer prevention, organized early detection, diagnosis, treatment and quality of life.
**European Commission [10]**	European NCD policy initiative	2022–2027	NCD prevention; risk factors; screening; care; equity	Provides the EU framework for action on major NCDs, including health determinants, cardiovascular disease, diabetes and chronic respiratory disease.

First author / issuing organization	Evidence type	Year / data basis	Analytical domain(s)	Main contribution to the synthesis
**European Commission [11]**	European cardiovascular policy framework	2025	Cardiovascular prevention; risk-factor management; policy alignment	Provides the European policy context used to assess alignment of Greek cardiovascular-prevention reforms.
**Hellenic Statistical Authority [17]**	National population health survey	2019; Greek population aged ≥15 years	Epidemiological burden; chronic conditions	Provides national self-reported prevalence estimates for chronic illness and specific chronic conditions used in the epidemiological assessment.
**OECD / European Commission [14]**	Country cancer profile	2025 report; cancer burden, prevention, early detection and cancer-care indicators	Cancer burden; screening; inequalities; surveillance	Provides Greece-specific cancer burden, risk-factor, early-detection and cancer-care information; the manuscript uses it for 2022 cancer incidence estimates, 2021 mortality comparisons and 2019 screening indicators.
**Hellenic Statistical Authority [18]**	National vital-statistics source	2023	Epidemiological burden; mortality	Provides observed national cause-of-death counts, including deaths from circulatory diseases and neoplasms.
**OECD [19]**	International comparative indicator report	2025; latest comparable official statistics available for individual indicators	Mortality; access; workforce; financial protection	Provides comparative indicators on population health and health-system performance across OECD countries.

First author / issuing organization	Evidence type	Year / data basis	Analytical domain(s)	Main contribution to the synthesis
**Athanasakis et al. [15]**	Greece-specific NCD policy/health-system assessment	2026	Primary care; workforce; coordination; screening follow-up; surveillance; governance	Provides recent evidence on barriers to earlier NCD intervention in Greece, including primary-care capacity, coordination, workforce and outcome-monitoring gaps.
**Hellenic Statistical Authority [20]**	National health-expenditure statistics	2024; System of Health Accounts	Financing and resource allocation	Provides national estimates of total health expenditure and its public/private financing structure.
**Eurostat [21]**	Methodological and analytical report	2016; health expenditure by diseases and conditions	Financing; disease-specific expenditure	Provides methodological and analytical evidence for examining health expenditure by disease and condition.
**Siskou et al. [22]**	Economic analysis	2016 publication; Greek disease-specific expenditure data	Financing; disease-specific expenditure	Provides estimates of health expenditure by disease, age and gender in Greece and contributes to the manuscript's historical disease-specific expenditure assessment.
**World Health Organization [12]**	International implementation framework	Implementation Roadmap 2023–2030	NCD prevention; surveillance; governance; health-system action	Provides the international framework for implementing the WHO Global Action Plan for NCD prevention and control.

First author / issuing organization	Evidence type	Year / data basis	Analytical domain(s)	Main contribution to the synthesis
**OECD [25]**	International patient-reported survey report	2025; first international PaRIS survey of primary-care patients aged ≥45 years, with emphasis on people with chronic conditions	Primary care; continuity; coordination; patient-reported outcomes and experiences	Provides patient-reported evidence on healthcare outcomes, experiences, continuity and coordination, including Greece-specific comparisons used in the manuscript.
**Jørgensen et al. [13]**	WHO Health Evidence Network evidence synthesis	2024; systematic screening programmes for cardiovascular risk factors and preclinical CVD	Cardiovascular screening; effectiveness	Synthesizes evidence on whether systematic population-level cardiovascular screening reduces disease burden and supports the manuscript's distinction between detection and demonstrated effectiveness.
**OECD / European Commission [24]**	European comparative analytical report	2026; European Cancer Inequalities Registry	Cancer care; screening; inequalities; international comparison	Provides cross-country evidence and policy analysis on high-value cancer care and inequalities across European health systems.
**European Observatory on Health Systems and Policies / OECD [16]**	European health-policy synthesis	2025; EU, Iceland and Norway	Prevention; primary care; digitalization; health-system reform	Synthesizes recent European reform trends, including primary prevention of NCDs, strengthening primary care and digital health.

Supplementary Table S2. Database search strategy

Database	Search strategy	Final search date
**PubMed/MEDLINE**	("Greece"[Title/Abstract] OR "Greek"[Title/Abstract]) AND ("non-communicable disease*"[Title/Abstract] OR NCD[Title/Abstract] OR NCDs[Title/Abstract] OR "chronic disease*"[Title/Abstract] OR "cardiovascular disease*"[Title/Abstract] OR cancer[Title/Abstract] OR	10 August 2026

Database	Search strategy	Final search date
	diabetes[Title/Abstract] OR "chronic obstructive pulmonary disease"[Title/Abstract] OR COPD[Title/Abstract] OR obesity[Title/Abstract]) AND (prevention[Title/Abstract] OR screening[Title/Abstract] OR "early detection"[Title/Abstract] OR "primary care"[Title/Abstract] OR "primary health care"[Title/Abstract] OR financing[Title/Abstract] OR "health expenditure"[Title/Abstract] OR surveillance[Title/Abstract] OR registr*[Title/Abstract] OR "quality of care"[Title/Abstract] OR governance[Title/Abstract] OR equity[Title/Abstract] OR inequalit*[Title/Abstract])	
**Scopus**	TITLE-ABS-KEY (Greece OR Greek) AND TITLE-ABS-KEY ("non-communicable disease*" OR NCD OR NCDs OR "chronic disease*" OR "cardiovascular disease*" OR cancer OR diabetes OR "chronic obstructive pulmonary disease" OR COPD OR obesity) AND TITLE-ABS-KEY (prevention OR screening OR "early detection" OR "primary care" OR "primary health care" OR financing OR "health expenditure" OR surveillance OR registr* OR "quality of care" OR governance OR equity OR inequalit*)	10 August 2026
**Web of Science Core Collection**	TS=(Greece OR Greek) AND TS=("non-communicable disease*" OR NCD OR NCDs OR "chronic disease*" OR "cardiovascular disease*" OR cancer OR diabetes OR "chronic obstructive pulmonary disease" OR COPD OR obesity) AND TS=(prevention OR screening OR "early detection" OR "primary care" OR "primary health care"	10 August 2026

Database	Search strategy	Final search date
	OR financing OR "health expenditure" OR surveillance OR registr* OR "quality of care" OR governance OR equity OR inequalit*)	
**CINAHL**	(TI Greece OR AB Greece OR TI Greek OR AB Greek) AND (TI "non-communicable disease*" OR AB "non-communicable disease*" OR TI NCD OR AB NCD OR TI "chronic disease*" OR AB "chronic disease*" OR TI "cardiovascular disease*" OR AB "cardiovascular disease*" OR TI cancer OR AB cancer OR TI diabetes OR AB diabetes OR TI "chronic obstructive pulmonary disease" OR AB "chronic obstructive pulmonary disease" OR TI COPD OR AB COPD OR TI obesity OR AB obesity) AND (TI prevention OR AB prevention OR TI screening OR AB screening OR TI "early detection" OR AB "early detection" OR TI "primary care" OR AB "primary care" OR TI "primary health care" OR AB "primary health care" OR TI financing OR AB financing OR TI "health expenditure" OR AB "health expenditure" OR TI surveillance OR AB surveillance OR TI registr* OR AB registr* OR TI "quality of care" OR AB "quality of care" OR TI governance OR AB governance OR TI equity OR AB equity OR TI inequalit* OR AB inequalit*)	10 Aug

Additional search methods: Targeted searches were conducted in official websites and repositories of the Greek Ministry of Health, Hellenic Statistical Authority (ELSTAT), National Public Health Organization (EODY), National Organization for Health Care Services Provision (EOPYY), Agency for Quality Assurance in Health (ODIPY), IDIKA/PROLAMVANO, WHO, OECD, Eurostat, the European Commission, the European Observatory on Health Systems and Policies, and European cancer-

information resources. Google Scholar and backward reference-list searching were also used to identify relevant grey literature, policy documents, programme reports and institutional sources. Searches combined terms related to Greece with NCD prevention, screening, primary care, financing, surveillance, registries, quality, governance, equity and health inequalities.
